# Molecular Genetic Characterization of an Anthrabenzoxocinones Gene Cluster in *Streptomyces* Sp. FJS31-2 for the Biosynthesis of BE-24566B and Zunyimycin Ale

**DOI:** 10.3390/molecules21060711

**Published:** 2016-05-30

**Authors:** Yuhong Lü, Changwu Yue, Meiyun Shao, Shengyan Qian, Ning Liu, Yuxin Bao, Miao Wang, Minghao Liu, Xiaoqian Li, Yinyin Wang, Ying Huang

**Affiliations:** 1 Guizhou Key Laboratory of Microbial Resources & Drug Development, Zunyi Medical University, Zunyi, Guizhou 563003, China; 18585737637@163.com (Y.L.); meiyun216@126.com (M.S.); qianshengyan@126.com (S.Q.); baoyuxin8680915@126.com (Y.B.); wangmiao_good@163.com (M.W.); xiaoqianli0908@163.com (X.L.); yinyinwang0530@126.com (Y.W.); 2 State Key Laboratory of Microbial Resources, Institute of Microbiology, Chinese Academy of Sciences, Beijing 100101, China; fussliu@126.com (N.L.); lysf1987313@163.com (M.L.)

**Keywords:** polyketide synthases, genome mining, genetic engineering, anthrabenzoxocinones, gene cluster

## Abstract

Genome mining is an effective tool used to discover novel natural products from actinomycetes. Genome sequence analysis of *Streptomyces* sp. FJS31-2 revealed the presence of one putative type II polyketide gene cluster (ABX), which may correspond to type II polyketide products including BE-24566B and its chloro-derivatives. The addition of natural humus acid successfully activated the biosynthsis of the abx gene cluster. BE-24566B and its chloro-derivatives, named zunyimycin A, were also detected. The targeted deletion of the polyketide skeleton synthesis genes such as abxp, abxk, and abxs was performed in the wild strain to identify the gene cluster for BE-24566B biosynthesis.

## 1. Introduction

Aromatic polyketides are an important class of natural products with various biological activities, including antibacterial [1,2], antitumor [3,4], and antiviral effects [5]. These polyketides are isolated from plants or microorganisms [6,7]. Actinomycete-derived aromatic polyketides are mainly synthesized by type II polyketide synthases (PKSs) [8,9]. Malonyl-CoA is exclusively used as the extender unit during type II polyketide biosynthesis [10,11]. Starter units vary in different aromatic polyketide biosynthesis pathways, leading to various polyketide backbones [12,13]. Enzymes, such as ketoreductases [14,15], oxygenases [16,17], cyclases [18,19], methyltransferases [20], halogenase [21,22], and glycosyltransferases [23], function on the skeleton to form aromatic structures or provide novel biological activities once the polyketide chains are formed by PKSs [24].

**Figure 1:**
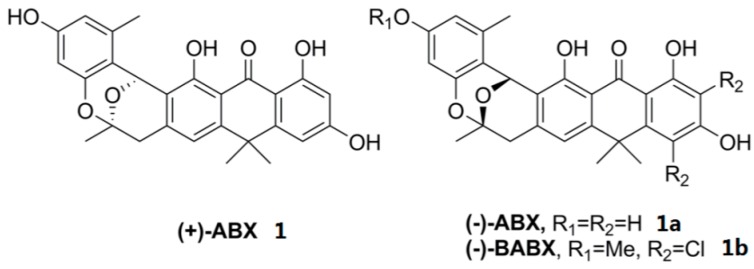
Chemical structure of ABXs.

Anthrabenzoxocinones (ABXs) are a group of hexacyclic aromatic ketones with bioactivities, such as antibacterial activity [25,26,27], anti-hypertensive activity [28], cholesterol-reducing activity [27] and nuclear hormone receptors [25]. ABX family members such as (+)-anthrabenzoxocinone ((+)-ABX) (BE-24566B, also named 1.264C,1), (−)-anthrabenzoxocinone ((−)-ABX)(1a), as well as (−)-bischloroanthrabenzoxocinone ((−)-BABX)(1b), (Figure 1) have been isolated from *Streptomyces* sp. (MA6657), *Streptomyces* sp. FXJ1.264, or *Streptomyces* *violaceusniger*, and *Actinomyces* sp. MA7150, respectively [29].

## 2. Results

### 2.1. Comparative Analysis of ABX Gene Clusters

**Figure 2:**
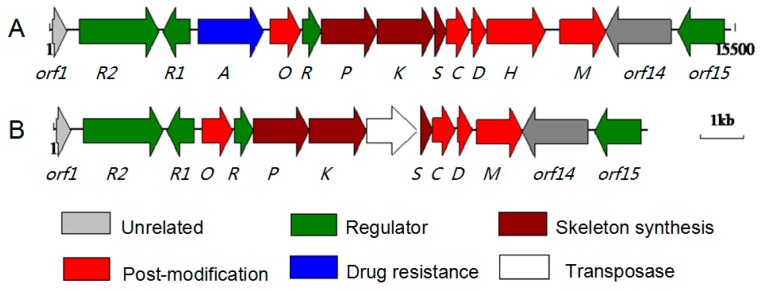
Organization of the ABX clusters from *Streptomyces* sp. FJS 31-2 (**A**) and *Streptomyces* sp. FXJ 1.264 (**B**).

The draft genome sequence of *Streptomyces* sp. FJS31-2 contains 11595310 bp, with a GC content of 70.73%. A total of 10877 putative protein coding sequences (CDSs) were identified in the draft genome covering 8840283 bp, accounting for 76.2% of the genome. Prediction of biosynthetic gene clusters on the whole genome level deduced by antiSMASH online reveals that the genome contains 23 secondary metabolism gene clusters such as polyketide synthase systems of every formally classified families (types I and II PKS), nonribosomal peptide synthetases (NRPS), PKS-NRPS hybrids, terpenes, siderophores, lantibiotics, aminocyclitol, melanin, ectoine, bacteriocin, and so on. As shown in Figure 2, genome sequencing and comparative analysis revealed that *Streptomyces* sp. FJS31-2 contains only one putative type II polyketide gene cluster (GenBank accession No. KU243130). This gene cluster shares a high similarity to its homologous gene cluster from *Streptomyces* sp. FXJ1.264 at the amino acid sequence (from 86.3% to 94.5%), gene content, and gene order levels (Table 1). Thirteen open reading frames are involved in the ABX polyketide backbone biosynthesis (abxp, abxk, and abxs), regulation (abxR2, abxR1, and abxR), drug resistance (abxA), cyclization (abxC and abxD), methylation (abxM), and halogenation (abxO and abxH) within the gene cluster of *Streptomyces* sp. 31-2 (Figure 2A). Ten homologous genes were found in *Streptomyces* sp. 1.264 (Figure 2B).

### 2.2. Characterization of the ABX Gene Cluster

**Figure 3:**
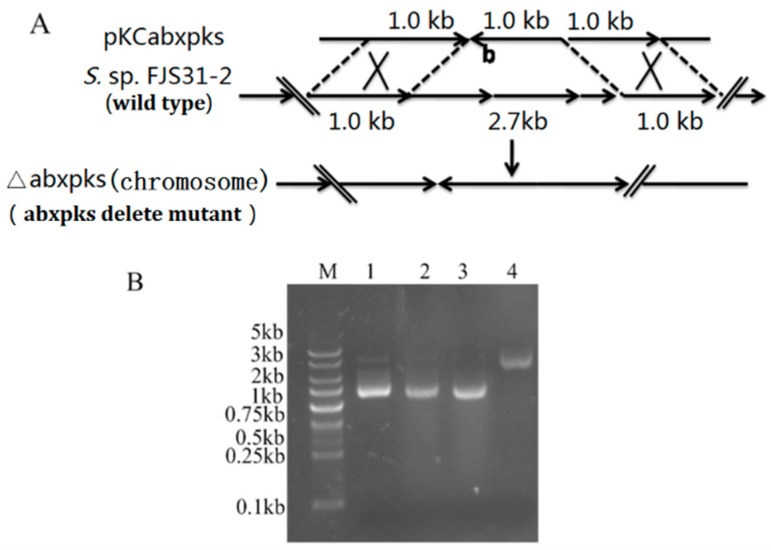
Deleted mutation of abxp, abxk, and abxs. (**A**) Mutation strategies of abxp, abxk, and abxs. (**B)** Analysis of abxp, abxk, and abxs deletion by PCR (lanes 1, 2, and 3: abxpks-deleted mutants; lane 4: wild-type strain; and M: DNA marker).

Results of PCR products sequencing of the three open reading fragments, namely, abxp, abxk and abxs, from the knockout mutant strain (△abxpks) indicated that the 2.7 kp DNA fragment was replaced by the kanamycin resistance gene (Figure 3).

**Figure 4:**
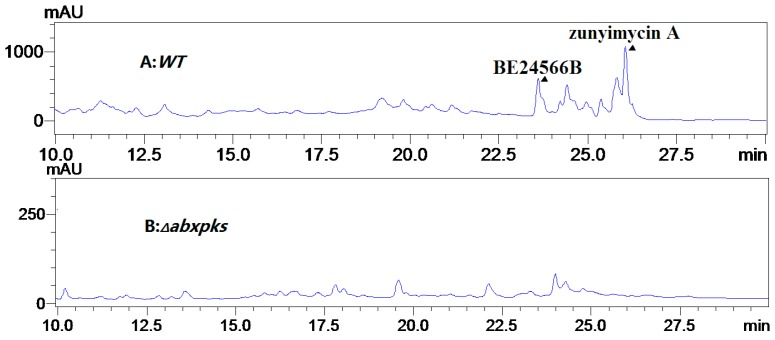
Metabolite profiling analysis of ABX biosynthesis. (**A**) Wild-type strain; (**B**) abxpks-deleted mutant strain.

Wild-type *Streptomyces* sp. FJS 31-2 and its abxpks-deleted mutant strain (△abxpks) were fermented, isolated, and chemically prepared under the same conditions, followed by high-pressure liquid chromatography (HPLC) detection. The result of the metabolite profiling analysis showed that the mutant ceased to produce BE-24566B. However, BE-24566B was still detected in the wild-type strain; this finding confirms that abxp, abxk, and abxs are involved in the biosynthesis of BE-2456B (Figure 4).

### 2.3. Chemical Identification of BE-24566B and Zunyimycin A

Chromatography and preparative high-pressure liquid chromatography (HPLC) yielded two compounds from strain *Streptomyces* sp. FJS 31-2. The NMR data of the two compounds were obtained in MeOD solvent. Compound **1** was obtained as a pale yellow powder. The ^13^C-NMR and DEPT spectra of **1** displayed signal for eighteen aromatic carbons at δC 167.7, 167.4, 159.6, 159.0, 157.0, 154.5, 152.4, 144.8, 138.0, 125.5, 119.5, 115.8, 113.2, 109.1, 108.4, 103.0, and 1012.7, which implied that the component **1** contained three benzene rings. Further, combining the signal of the carbonyl group at δC 192.8, the methane group at δC 35.0, the quaternary carbon at δC 35.0, and the carbon proton at δC 99.7 indicated that Compound **1** was BE-24566B. Except for the above information, the ^1^H-NMR spectra of **1** displayed signal at 13.5 (1H, s), 12.8 (1H, s), 7.16 (1H, s), 6.75 (1H, *d*, *J* = 2.0 Hz), 6.33 1H, *d*, *J* = 2.1 Hz), 6.27 (1H, *d*, *J* = 2.0 Hz), 6.16 (1H, *d*, *J* = 2.2 Hz), 2.52 (3H, s), 1.95 (3H, s), 1.88 (3H, s), 1.64 (3H, s), 3.24 (1H, *d*, *J* = 18.0Hz), and 3.24 (1H, *d*, *J* = 18.0Hz). This also demonstrated that the constituent was ABX.

Compound **2** was obtained as a pale yellow powder. The IR spectrum of **2** showed absorption at 3385 cm^−1^ and 1608 cm^−1^, indicative of the presence of hydroxyl and carbonyl groups, and the absorption at 1421 cm^−1^ and 1384 cm^−1^ illustrated the existence of a benzene ring. Its HR-TOF-MS ion at *m*/*z* 528.0714 [M]^−^ indicated the molecular formula to be C_27_H_22_Cl_2_O_7_, implying sixteen degrees of unsaturation, respectively. The ^13^C-NMR and DEPT spectra of **2** displayed signal for eighteen aromatic carbons at δC 164.5, 163.0, 156.9, 153.2, 152.6, 150.6, 148.3, 141.9, 133.4, 122.1, 117.7, 115.4, 112.6, 109.9, 107.6, 102.6, and 101.6, which implied the component **2** contained three benzene rings. Further, combining the signal of the carbonyl group at δC 190.8, the methane group at δC 39.6, the quaternary carbon at δC 39.5, and the carbon proton at δC 98.2 indicated that Compound **2** had the skeleton of BE-24566B. Except for the above information, the ^1^H-NMR spectra of **2** displayed signal at 7.01 (1H, s), 6.45 (1H, s), 6.27 (1H, s), 6.24 (1H, s), 2.52 (3H, s), 1.95 (3H, s), 1.88 (3H, s), 1.64 (3H, s), 3.24 (1H, *d*, *J* = 18.0Hz) and 3.24 (1H, *d*, *J* = 18.0). This also demonstrated the presence of BE-24566B.

**Figure 5:**
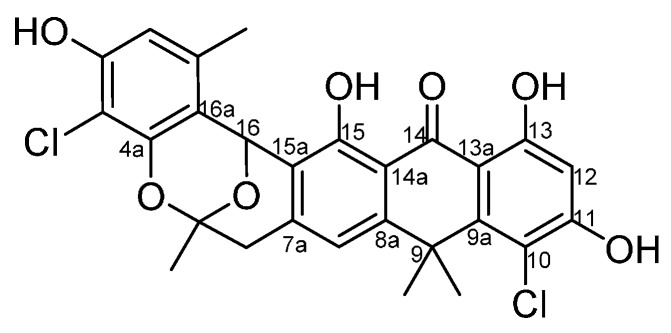
The structure of zunyimycin A.

The HMBC experiment showed a correlation between the proton at δH 7.01 and carbons at δC 39.6, 39.5, 109.9, and 122.1, which indicated that one hydroxyl atom connected with the C-8 position. The signal at δH 6.45 (1H, s), the carbons at δC 164.5, 112.6, and 164.5, and, combined with the ROSEY spectra, the δH 6.45 (1H, s) had no correlation, which implied that the signal at δH 6.45 connected with a benzene ring at position C-12. The proton signal at δH 6.27 (1H, s) correlated with δC 152.6, 150.6, and 115.4, which indicated that the signal at δH 6.27 connected with a benzene ring at position C-4. The proton signal at δH 6.24 (1H, s) had a key correlation with the signal at δC 98.2, which indicated that the signal at δH 6.24 connected with a benzene ring at position C-16. According to the above information, the relative structure of **2** was substituted by chlorine atoms at the C-2 and C-10 positions of BE-24566B; therefore, Compound **2** was named zunyimycin A (Figure 5).

## 3. Discussion

**Figure 6:**
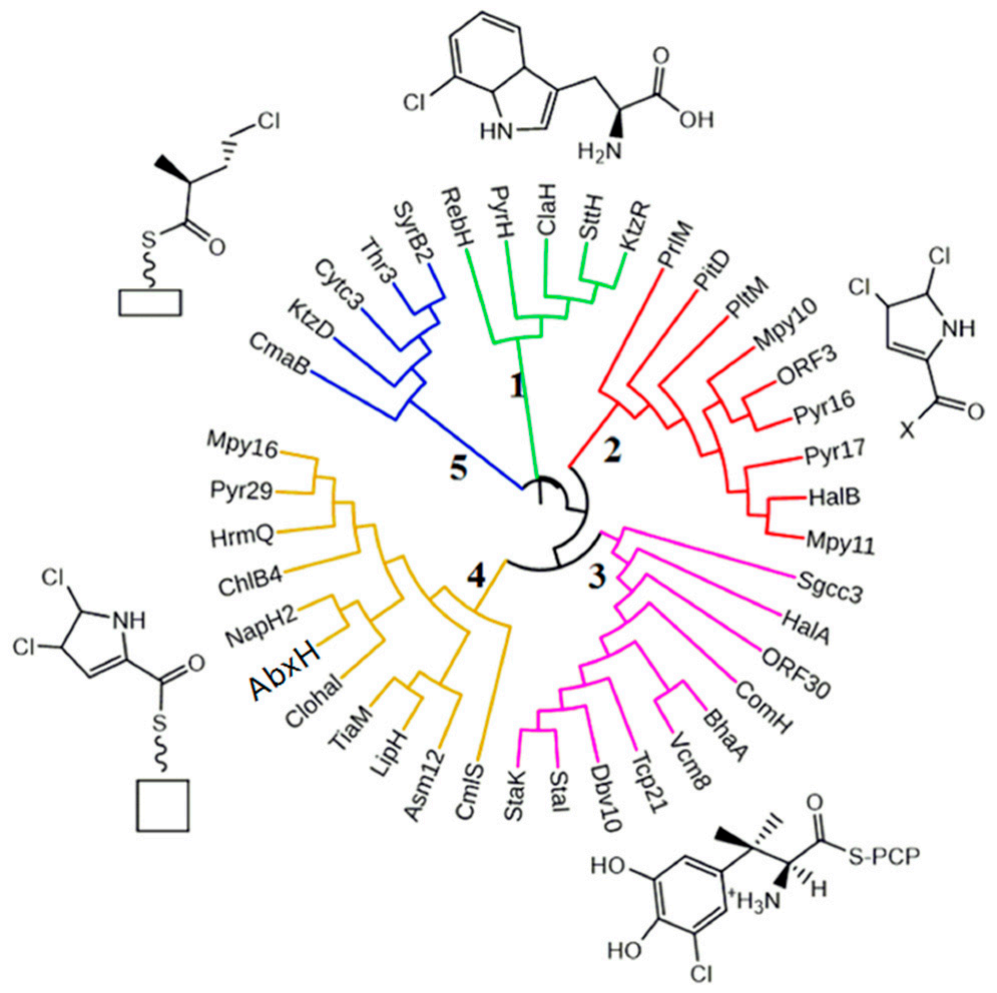
Phylogenetic analysis of identified halogenases from actinomycetes.

**Scheme 1:**
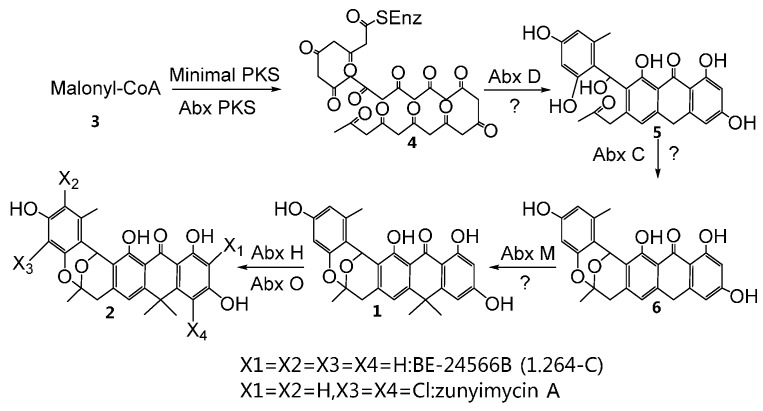
Proposed biosynthetic pathway of zunyimycin A.

Cluster analysis results show that AbxH is clustered with the identified halogenases involved in the halogenated modification of pyrrole or its derivatives. Furthermore, AbxH exhibits the highest similarity (*i.e*., 49% at the amino acid sequence level) to NapH2 (Figure 6), which is involved in the halogenation reaction of napyradiomycin [29]. These results suggest that AbxH may possess a capacity for the halogenation reaction of polyketide. No abxH homologous gene was predicted in the genome, and no halogenated natural product was detected from the fermented products of *Streptomyces* sp. 1.264; hence, abxH (halogenase) may play a vital role in zunyimycin biosynthesis (Scheme 1).

## 4. Materials and Methods

### 4.1. Strains, Plasmids, and Reagents

*Streptomyces* sp. FJS31-2 was isolated from a soil sample collected from the Fanjing Mountain of the Guizhou Province at an altitude of 800 m in October 2012. The strain was deposited in the China General Microbiological Culture Collection Center under accession number CGMCC 4.7321; the strain exhibits biological activities against *Candida albicans*, *Bacillus subtilis*, *Staphylococcus aureus*, and *Micrococcus luteus*. The nucleotide sequencing was performed by BGI (Shenzhen, China) using an Illumina/Hiseq 2000 sequencer. Assembly was performed using SOAP *de novo* software [30]. Genomic DNA was directly extracted from the expanded culture of the ISP 2 liquid medium and used for genome sequencing and genomic library construction [31]. The bacterial strain EPI300-T1^R^ and the plasmid pCC2FOS were obtained from Epicentre (Madison, WI, USA) and used to construct a genomic library by using CopyControl™ HTP Fosmid Library Production Kit (Cat. No. CCFOS059; Sinopharm Chemical Reagent Co., Ltd. (Shanghai, China). Cloning plasmids, namely, pMD18-T and pSIMPLE-18 *Eco*R V/BAP, were purchased from Takara Biotechnology Company (Dalian, China) and used to construct a sub-clone library. Restriction enzymes, T4 DNA ligase, DNA end blunting kination ligation, and PCR kits were also purchased from the same company. The primers used for gene cloning and DNA sequencing were synthesized by Beijing Invitrogen Biotechnology Co., Ltd. (Beijing, China). The DNA gel extraction kit was obtained from Axygen Biosciences, Inc. (Hangzhou, China).

### 4.2. DNA Isolation, Manipulation, and Sequencing

Genomic DNA was isolated from *Streptomyces* sp. FJS31-2 by using the standard method described by Hopwood [32]. Molecular manipulation was conducted according to the standard methods described by Sambrook and Russel [33]. Plasmid and fosmid sequencing was performed on the 3730xl DNA analyzer at Beijing Invitrogen Biotechnology Co., Ltd. by using the shotgun cloning strategy. The pCC2/pEpiFOS forward and reverse primers (sequences in the CopyControl protocol) were used to sequence the fosmid ends. Moreover, the M13 primers were used to sequence the subclone ends. Draft genome sequencing of *Streptomyces* sp. FJS31-2 was performed on Illumina HiSeq2000 (BGI Biotechnology Co., Ltd. Shenzhen, China) by using a proprietary reversible terminator-based strategy.

### 4.3. The Genomic Library and Subclone Library Construction and Screening

The full-length sequence of the predicted lobophorin biosynthetic gene cluster was obtained by constructing the genomic library of *Streptomyces* sp. FJS31-2 in CopyControl pCC2FOS according to the manufacturer′s instructions. The library contains more than 6000 clones with approximately 40 kb inserted genomic DNA fragments. The genomic library was screened through colony PCR with primer pairs designed based on the nucleic acid sequences of abxH according to the results of the predictive analysis of the draft genome sequencing. The end of the positive clone was sequenced to confirm that the clone was located in the abx cluster. Next-round screening was performed with primer pairs designed based on the end sequences of the last round of screening results until three positive clones overlapping one another were obtained. Accordingly, 1 μg of fosmid DNA was fragmented using an ultrasonic cell disruptor under 200 W for 1 s on ice. DNA fragments within 2–4 kb size were recovered using the AxyPrep DNA gel extraction kit. The fragments were cloned into pSIMPLE-18 *Eco*R V/BAP vector to construct subclone libraries after being end blunted and 5′-phosphorylated. PCR screening was performed using sequence-specific primers designed based on the end sequences of each gap. The positive clones were then sequenced with M13-47F/M13-48R and specific primers.

### 4.4. Analysis of the Anthrabenzoxocinone Biosynthetic Gene Cluster

A 16.5-kb contiguous DNA segment was obtained by subcloned library sequencing and deposited in GenBank under accession number KU243130. The sequence was uploaded to the antiSMASH website [34] and used to determine gene content and order. The open reading frames proposed for the biosynthesis pathway of the anthrabenzoxocinone skeleton, post modification, and regulation were deduced based on functional gene analysis. The analysis was performed by comparing the sequence homology with its homologous clusters by using BlastP. These clusters were found by antiSMASH. The comparative analysis and the global rearrangement structure of the two anthrabenzoxocinone biosynthetic gene clusters were conducted using Mauve software [35].

### 4.5. Inactivation of the Anthrabenzoxocinone Biosynthetic Gene Cluster

A 2.7-kb DNA fragment (from 6165 nt to 8872 nt of KU243130) encoding the ABX polyketide backbone biosynthesis (*abxp*, *abxk*, and *abxs*) was replaced by the kanamycin resistance protein-coding region (AAF85969.1). This region was constructed by PCR, inserted into the pKC1139 plasmid, and transformed into *E. coli* ET12567. The constructs for gene inactivation were introduced into *Streptomyces* sp. FJS31-2 by conjugal transfer following the procedure of Hopwood^2^. Disruption was confirmed by PCR analysis. HPLC (LC20AT, Shimadzu, Beijing, China) was used to confirm the abolishment of anthrabenzoxocinone production.

### 4.6. Fermentation, Isolation, and Chemical Identification of BE-24566B and Zunyimycin A

*Streptomyces* sp. FJS31-2 was cultured using 140 × 500 mL shake flasks containing 100 mL of ISP 2 agar medium with 10% natural humus acid water extracts and then incubated for 15 d at 28 °C. The solid culture was mashed and extracted three times with 140 L of ethanol after cultivation. The organic portion was then concentrated *in vacuo* to remove the solvent. The crude extract was applied to silica gel column chromatography using the CHCl_3_/MeOH gradient to obtain the crude products. Further purification was conducted using Sephadex LH-20 (GE Healthcare, Tokyo, Japan) (MeOH) column and RP-HPLC (Shimadzu SPD-M20A with Xbridge ODS 10 mm × 150 mm column). Compounds were identified with an HRESI-MS (Waters Xevo G2 QTOF mass spectrometer (Waters corportion, Milford, MA, USA) and NMR (Bruker AV 600 MHz) (Bruker Corporation, Karlsruhe, Germany) analyses.

## 5. Conclusions

In summary, a cryptic anthrabenzoxocinone gene cluster was identified by genome mining. This cluster was successfully activated by adding natural humus acid. Novel halogenated natural products were isolated, characterized, and named as zunyimycin A. Furthermore, the results indicated that halogenase AbxH is responsible for the halogenation reaction of zunyimycin A and may provide potential for the halogenation of natural products.

## Figures and Tables

**Table 1:** Homologous analysis of abx gene cluster from *Streptomyces* strains.

*S.* sp. FJS31-2		*S.* sp. FXJ1.264		Identit %	Deduced Function
Protein	Size aa	Protein	Size aa		
Orf1	87	1.264DNAGM006278	87	93	Hypothetical protein.
		1.264DNAGM006279	319		Cation efflux family
AbxR2	616	1.264DNAGM006280	616	91	SARP transcriptional regulator
AbxR2	207	1.264DNAGM006281	219	88	TetR transcriptional regulator
AbxA	490				MFS transporter
AbxO	227	1.264DNAGM006282	231	90.5	Monooxygenase
AbxR	141	1.264DNAGM006283	141	93.6	AraC transcriptional regulator
AbxP	421	1.264DNAGM006284	421	96	Beta-ketoacyl_synthase, t2PKS
AbxK	426	1.264DNAGM006285	424	90.3	Beta-ketoacyl_synthase, t2PKS
		1.264DNAGM006286	394		Transposase
AbxS	85	1.264DNAGM006287	88	86.4	Acyl carrier protein
AbxC	167	1.264DNAGM006288	168	86.3	cyclase/dehydrase
AbxD	113	1.264DNAGM006289	113	88.5	Polyketide synthesis cyclase
AbxH	436				tryptophan halogenase
AbxM	343	1.264DNAGM006290	349	91.4	*O*-methyltransferase
Orf14	491	1.264DNAGM006291	490	91.0	Lipase maturation factor
Orf15	348	1.264DNAGM006292	348	94.5	LacI transcription regulator
